# Potential Health Benefits of Banana Phenolic Content during Ripening by Implementing Analytical and In Silico Techniques

**DOI:** 10.3390/life13020332

**Published:** 2023-01-25

**Authors:** Eftichia Kritsi, Thalia Tsiaka, Georgios Sotiroudis, Elizabeth Mouka, Konstantinos Aouant, Georgia Ladika, Panagiotis Zoumpoulakis, Dionisis Cavouras, Vassilia J. Sinanoglou

**Affiliations:** 1 Laboratory of Chemistry, Analysis & Design of Food Processes, Department of Food Science and Technology, University of West Attica, Agiou Spyridonos, 12243 Egaleo, Greece; 2 Institute of Chemical Biology, National Hellenic Research Foundation, 48, Vas. Constantinou Ave., 11635 Athens, Greece; 3 Department of Biomedical Engineering, University of West Attica, Agiou Spyridonos, 12243 Egaleo, Greece

**Keywords:** antioxidant and antiradical capacity, banana fruit, carbonic anhydrase enzymes, in silico tools, liquid chromatography-mass spectrometry (LC-MS/MS), phenolic compounds, potential health benefits ripening

## Abstract

Banana ranks as the fifth most cultivated agricultural crop globally, highlighting its crucial socio-economic role. The banana’s health-promoting benefits are correlated with its composition in bioactive compounds, such as phenolic compounds. Thus, the present study attempts to evaluate the potential health benefits of banana phenolic content by combing analytical and in silico techniques. Particularly, the total phenolic content and antioxidant/antiradical activity of banana samples during ripening were determined spectrophotometrically. In parallel, liquid chromatography-tandem mass spectrometry (LC-MS/MS) analysis was implemented to unravel the variations in the phenolic profile of banana samples during ripening. Chlorogenic acid emerged as a ripening marker of banana, while apigenin and naringenin were abundant in the unripe fruit. In a further step, the binding potential of the elucidated phytochemicals was examined by utilizing molecular target prediction tools. Human carbonic anhydrase II (hCA-II) and XII (hCA-XII) enzymes were identified as the most promising targets and the inhibitory affinity of phenolic compounds was predicted through molecular docking studies. This class of enzymes is linked to a variety of pathological conditions, such as edema, obesity, hypertension, cancer, etc. The results assessment indicated that all assigned phenolic compounds constitute great candidates with potential inhibitory activity against CA enzymes.

## 1. Introduction

Nowadays, consumers have incorporated in their dietary habits foods which not only cover their nutritional demands but also confer various health benefits. The banana (*Musa* sp.) fruit is recorded as one of the most economically important food sources and it is ranked as the fourth most cultivated crop, following rice, wheat, and maize in a worldwide level [1,2]. Banana is characterized as a low-cost food, providing high levels of energy because of its high composition in starch [3]. 

According to the Food and Agriculture Organization (FAO) of the United Stated (https://www.fao.org/home/en/) (accessed on 1 December 2022), the average banana production and trade has been expanded from 69 million tons in 2000–2002 to around 121 million tons in 2020–2021, highlighting its remarkable increase in the last two decades [4,5]. Recent statistics [4,5] indicates that India, China, Philippines, Ecuador, and Brazil are the world’s top banana producers. 

The banana fruit consists of two different parts, including the pulp which is the consumable portion of the fruit and the peel which is generally considered as a byproduct [6]. It is well-known that both banana flesh and skin constitute valuable raw materials, comprising a rich source of various bioactive compounds. Banana fruit is composed especially of phenolic compounds, carotenoids, flavonoids, biogenic amines, phytosterols, and other phytochemicals [7,8,9]. Compared to various berries, herbs, and vegetables, banana presents higher antioxidant potential ascribed to the high contents of the described constituents [10]. In addition, the study of bananas’ carotenoids content reveal that α-carotene, β-carotene, and β-cryptoxanthin present significant provitamin A activity, while lycopene and luteolin possess important antioxidant capacity [6,11]. 

Banana pulp contains a plethora of phenolic compounds, such as catechin, epicatechin, lignin, tannin, and anthocyanin [12]. Particularly, recent studies have identified several phenolic compounds, including epicatechin, gallocatechin, and a variety of phenolic acids (i.e. ferulic, sinapic, salicylic, gallic, p-hydroxybenzoic, vanillic, syringic, gentisic, protocatechuic, and p-coumaric acids), as the major components contained in banana [3,13,14]. 

According to latest research results, the phenolic constituents of bananas could be denoted as indicators of the ripening stage, which, in general, is tightly associated with the total phenolic content, antioxidant, and antiradical activities of both unripe and ripe bananas [15,16,17]. In addition, a recent study attempted to characterize and quantify the phenolic compounds in a variety of ripe and unripe banana samples, by implementing several analytical techniques [6]. The evaluation of the derived results revealed that epicatechin, catechin, kaempferol (flavonoids), and caftaric, caffeic, syringic, chlorogenic, gallic, and protocatechuic acids (phenolic acids) were present in great amounts, highlighting the correlation between the phenolic structures and the banana’s antioxidant activity [6]. Moreover, it is also well-known that the aforementioned compounds exhibit antiviral, antithrombotic, antiallergenic, anti-inflammatory, antibacterial, anti-diabetic, anti-cancer, and vasodilatory properties [18,19,20]. As a result, banana consumption is causally linked with health-promoting benefits and in general with human well-being.

Hence, the present study aims to combine analytical and in silico techniques, in an effort to correlate the phenolic content of banana with potential health-promoting effects. Therefore, the first step of our pipeline included the evaluation of the total phenolic content and the antioxidant/antiradical activity of banana samples at different ripening stages. Alongside, LC-MS/MS analysis was carried out in order to elucidate the phenolic profile of the examined banana extracts. Furthermore, the recorded phenolic compounds were tested against a variety of molecular targets, utilizing in silico tools, to discover the most promising molecular targets. Finally, molecular docking studies were performed to scrutinize the inhibition potential of the phenolic compounds in relation to the proposed molecular targets (hCA-II and hCA-XII), underscoring their capacity as future inhibitors targeting various pathological conditions (edema, glaucoma, cancer, hypertension, etc.). 

## 2. Materials and Methods

### 2.1. Standards and Reagents

All solvents used for the analytical and spectrophotometric assays were obtained from Mallinckrodt Chemical Works (St. Louis, MO, USA) and were of HPLC grade. Folin–Ciocalteu phenol reagent, Trolox (6-hydroxy-2,5,7,8-tetramethylchroman-2-carboxylic acid), Ferrous sulfate heptahydrate, 2,4,6-tris(2-pyridyl)-S-triazine (TPTZ), and iron(III) chloride hexahydrate were obtained from the Sigma Chemical Co. (St. Louis, MO, USA); ABTS (2,20-azinobis (3-ethylbenzothiazoline-6-sulfonic acid)) from the Tokyo Chemical Industry Co. Ltd. (Tokyo, Japan); and gallic acid (3,4,5-trihydroxybenzoic acid) from Alfa Aesar (Karlsruhe, Germany). 

Regarding LC-MS/MS experiments, all solvents were of LC-MS grade. Specifically, acetonitrile, methanol, water, and formic acid were obtained by Chem-Lab (Zedelgem, Belgium), Merck KGaA (Darmstadt, Germany), Thermo Fischer Scientific (Waltham, MA, USA), and LGC Promochem (Teddington, UK), respectively. The phenolic compounds (+)-kaempferol, quercetin, eriodictyol, taxifolin, gallic acid, rosmarinic acid, o-coumaric, and caffeic acid were purchased from Extrasythese (Genay, France). Naringenin, hesperitin, salicylic acid, syringic acid, and resveratrol were procured from Alfa Aesar (Ward Hill, MA, USA). Phenolic standards of vanillin, ethyl vanillin, chlorogenic acid, coumaric acid, m-coumaric acid, (-)-catechin, protocatechuic acid, pyrocatechol, syringaldehyde, p-hydroxy-benzoic acid, benzoic acid, apigenin, myricetin, and ferulic acid were obtained from Sigma-Aldrich (St. Louis, MO, USA).

### 2.2. Sample Collection 

The banana samples, belonging to the Cavendish variety (Musa acuminata), were delivered from Ecuador and were kindly donated from Pefanis Company (https://www.pefanis.com.gr/en/) (accessed on 1 December 2022), which is based in Athens, Greece. A controlled atmosphere ripening room, located at the company’s facilities, was used to activate the maturation of green bananas artificially on day 0. A day later (day 1), twelve bananas hands consisting of 7 or 8 fingers per hand, with no visual defects, were randomly picked out from banana bags placed in the ripening room and transferred to the laboratory. The banana samples were then placed in an oven with a controlled temperature of 18.0 ± 0.5 °C and a relative humidity of 60 ± 2%, in order to evaluate the banana ripening stages. All experiments were applied over a period of 21 days during fruit ripening (days 2, 4, 7, 9, 11, 14, 17, and 21) and eight biological replicates of bananas were randomly selected for the measurements. 

### 2.3. Extraction of Phenolic Compounds and Spectrophotometric Assays

For the spectrophotometric assays, about 3 g of banana flesh was diluted in aqueous methanol (80% *v*/*v*) in a mass-to-volume ratio 1:5 (*w*/*v*). The solutions were stored in closed flasks for 24 h, at room temperature (20 °C). Then, the extracts were filtered by a Buchner funnel and the filtrates were diluted in 20.00 mL with aqueous methanol and stored at 4 °C for further spectrophotometric analysis. All spectrophotometric measurements were performed in triplicate with a Spectro 23, Digital Spectrophotometer (Labomed, Inc., Los Angeles, CA, USA). The total phenolic content (TPC) of banana flesh extracts was determined by applying a modified micromethod of Folin–Ciocalteu colorimetric assay, by Andreou et al. [21]. Measurements were performed at 750 nm. The results were expressed as mg of gallic acid equivalents (GAE) per 1 g of banana flesh, using standard solutions with a range of 20–500 mg·L^−1^ gallic acid. The antiradical activity on ABTS^●+^ radical of banana flesh extracts was determined according to the method described by Lantzouraki et al. [22]. Absorbance was measured at 734 nm. Trolox was used as standard compound and the antiradical activity of samples was expressed as mg of Trolox Equivalents (TE) per 1 g of banana flesh, using standard solutions with a concentration range of 0.20–1.5 mM. The Ferric Reducing Antioxidant Power (FRAP) assay of banana flesh extracts was determined according to the modified method of Lantzouraki et al. [23]. Absorbance was measured at 595 nm. A standard curve was prepared using various concentrations (600–2000 μM) of FeSO_4_·7H_2_O stock solutions. The results were expressed as mg of Fe (II) per 1 g of banana flesh. 

### 2.4. LC-ESI(−)-MS/MS Analysis

The phenolic compounds in the banana extracts were elucidated by using an in-house developed library, which included 26 reference standards [24]. An Agilent 1200 HPLC system (Agilent Technologies, Santa Clara, CA, USA) coupled with a 3200 Q TRAP triple-quadrupole linear ion trap mass spectrometer (Sciex, Framingham, MA, USA) was used for the analysis.

An Agilent Eclipse Plus C-18 reversed-phase column (50 mm × 2.1 mm inner diameter, 3.5 µm particle size) combined with a RRLC in-line filter kit (2.1 mm, 0.2 µm filter) (Agilent Technologies, Santa Clara, CA, USA) was used for the chromatographic separation. The column and the autosampler temperature were adjusted at 25 °C. Water-0.2% *v*/*v* formic acid (Solvent A) and acetonitrile-0.1% *v*/*v* formic acid (Solvent B) consisted the mobile phase. The gradient elution program (Table 1) applied for the separation of the phenolic standards was developed in previous works of our group [25].

Aliquots of 1.5 mL of banana extracts were dried and the dried residues were diluted at 1000 μL of methanol-0.1% formic acid (injection solvent). The injection volume was 5 μL. Samples were filtered through Chromafil Xtra PET 0.45 μm (Macherey-Nagel, Düren, Germany).

The MS/MS source was electrospray ionization (ESI) source and the ionization of the phenolic compounds was performed in negative ion mode. The identification of phenolics in banana extracts was conducted by enhanced mass spectrum (EMS) survey scans and information dependent acquisition (IDA)-triggered MS/MS scans (EPI—enhanced product ion scans). The conditions of EMS and IDA-EPI experiments are described in Supplementary data (Table S1). The mass tolerance for MS/MS analysis was set at 5 ppm.

The LC-MS/MS data were processed with Analyst software (version 1.4.2) (Sciex, Framingham, MA, USA). All samples were measured in duplicate and one-way ANOVA (Statistica package, trial version 12, TIBCO Software Inc., Palo Alto, CA, USA) was applied for the statistical analysis of the samples. All measurements were carried out at 95% confidence level (*p*-value ≤ 0.05).

### 2.5. Computational Studies

#### 2.5.1. Phenolic Compounds Preparation 

The phenolic compounds, apigenin, caffeic acid, chlorogenic acid, kaempferol, naringenin, quercetin, rosmarinic acid, and syringic acid contained in banana pulp samples and characterized by LC-ESI(-)-MS/MS analysis, were sketched in 2D format (.SMILES) and prepared at pH = 7.0 ± 0.5, by applying LigPrep program [26] of MAESTRO interface [27].

#### 2.5.2. Protein Target Prediction and Molecular Docking Studies 

In continuation, the compounds were subjected to potential target prediction, using the freely accessible web-based prediction tools Random Forest QSAR (http://rfqsar.kaist.ac.kr/home.php) (accessed on 1 December 2022) and Swiss Target Prediction (http://www.swisstargetprediction.ch/) (accessed on 1 December 2022). The Random Forest QSAR tool [28] supports target identification based on structure–activity relationships (SARs) of tested molecules and the Swiss Target Prediction tool [29] estimates the most probable macromolecular targets of a small molecule by combining its 2D and 3D similarity with a library of 370,000 known actives on more than 3000 proteins. 

The results assessment, based on the probability values (Table S2, Supplementary Materials), indicated human Carbonic Anydrase II (hCA-II) and XII (hCA-XII) as common and relevant molecular targets for all examined phenolics. The crystal structures of hCA-II (PDB ID: 5AML) and hCA-XII (PDB ID: 5MSA) were downloaded from the Protein Data Bank (PDB) and were acquired for protein preparation [30]. The preparation, especially, included the removal of all water molecules, the addition of missing residues and hydrogen atoms, and the production of a geometrically stable structure, by performing energy minimization with the force field OPLS3. The similarity between the superimposing crystallographic and docking poses of the co-crystallized ligands was utilized for the validation process. The RMSD values derived from the superimposition of the docked and co-crystallized poses at the binding site of hCA-II and hCA-XII are 1.50 and 1.48, respectively. 

Finally, a grid box with dimensions 10 × 10 × 10 Å was generated and the favorable binding poses of the examined phytochemicals were predicted, by applying the Glide Standard Precision (SP) mode [31]. The maximum number of docking poses was 10 and each of them was visually inspected and analyzed. 

### 2.6. Statistical Analysis

Statistical analysis of the variation of the banana image features and physicochemical parameters between ripening days was performed by means of the non-parametric Mann–Whitney–Wilcoxon test for two classes. The Python scipy.stats library (https://docs.scipy.org/doc/scipy/tutorial/stats.html, accessed on 1 December 2022) was used. The pairwise correlation matrix was formed among the physicochemical parameters and was computed by the use of Python’s pandas library (https://pandas.pydata.org/docs/reference/api/pandas.DataFrame.corr.html, accessed on 1 December 2022). Moreover, results were analyzed using a significance level of *p* < 0.05 with one-way ANOVA and post hoc analysis. These calculations were carried out using SPSS (IBM SPSS Statistics. version 29.0. Chicago, IL USA) for Windows.

## 3. Results and Discussion

### 3.1. Spectrophotometric Assays in Banana Samples

**Figure 1:**
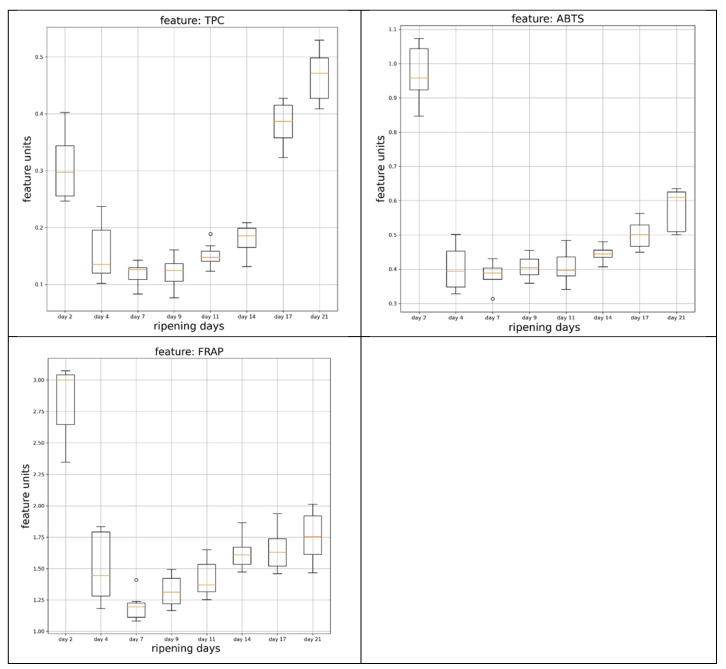
Total phenolic content (TPC), antiradical activity (ABTS), and antioxidant activity (FRAP) of banana flesh samples during storage at 18.0 ± 0.5 ^°^C.

The total phenolic content and the antiradical and antioxidant activity of banana flesh samples at days 2, 4, 7, 9, 11, 14, 17, and 21 of storage were estimated using the Folin–Ciocalteu, ABTS, and FRAP spectrophotometric methods, and are shown in Figure 1 and Table S3. 

The results revealed that phenolic content as well as the antiradical and antioxidant activity were dependent on the banana-ripening stage. Specifically, total phenolic content and antiradical activity showed a significant (*p* < 0.05) decrease on day 4 of the experiment; thence, insignificant variations until day 11 and afterwards showed a gradual and significant (*p* < 0.05) increase until the end of the storage period. Furthermore, the antioxidant activity of banana flesh presented an initial significant (*p* < 0.05) decrease at day 4, an increase at day 7, stabilization until day 11, and a significant progressive increase until the end of the storage period. High positive correlation (*p* < 0.05) was determined between antiradical and antioxidant activity (0.95) whereas total phenolic content correlated positively with antiradical and antioxidant activity (0.53 and 0.52, respectively) (Table 2).

In accordance with the results of the present study, many researchers reported that green bananas have lower total phenolic content than ripen fruits [32,33,34,35].

### 3.2. Phenolic Profile of the Examined Banana Samples by LC-MS/MS Analysis

The retention time and spectra characteristics of phenolic compounds, which were tentatively elucidated in the banana extracts at different days (days 2, 4, 7, 9, 11, 14, 17, and 21), are displayed in Table 3. The chromatographs and mass spectra of indicative detected compounds in bananas extracts are illustrated in Figure S1.

The processing and interpretation of LC-MS/MS data revealed the presence of flavonoids naringenin and kaempferol in the samples of all days, while the phenolic acids, chlorogenic and caffeic acid, were detected as the ripening proceeded (days 17 and 21). Furthermore, syringic acid was absent only at the beginning (day 2) and at the end (day 21) of the storage period. On the other hand, apigenin and quercetin were identified in the samples of certain days, while rosmarinic acid was observed at day 2 (first day of the ripening process) and in the middle of the storage period (days 9 and 11). In particular, apigenin was determined in the first stages of ripening, whilst quercetin was found at the end of this process. The presence of the elucidated phenolic compounds during different days of ripening is reported in Table 4.

According to literature data, banana flesh contains various natural antioxidants, such as phenolics and vitamins. The main phenolic compounds detected in banana peel and flesh were flavonoids such as catechin, epicatechin, quercetin, myricetin, kaempferol, and cyanidin, phenolic acids such as ferulic, chlorogenic, p-hydroxybenzoic, vanillic, syringic, and p-coumaric acid and other phenolics such as lignin and tannins [6,18,36,37].

Focusing on the alterations in the content of naringenin and kaempferol, which were present throughout fruit ripening, essential variations were noticed. Kaempferol content was decreased in the first two experimental periods (from day 2 to day 4), then showed an important (*p*-value ≤ 0.05) increase at day 7, followed by a steady decrease from day 9 until day 17. However, at day 21 the content of kaempferol showed an exponential rise. The abovementioned outcome could be attributed to the degradation of various kaempferol derivatives, which led to the overall increase of the free form of kaempferol [19,38]. On the other hand, naringenin also exhibited a decrease from day 2 to day 4, and then its content did not vary significantly until the last three experimental days (day 14 and day 21), where it reduced gradually. This finding could be related to the parallel significant increase of kaempferol in bananas during ripening, since the aglycone form of kaempferol is derived from the free form of naringenin [39]. In addition, apigenin, which was present at the beginning and at the middle of ripening (Table 4), showed higher content when the bananas were fresh (day 2), a result which comes in agreement with the findings of Kongkachuichai et al., (2010), where apigenin was an indicative flavonoid of the fresh fruit [15]. 

**Figure 2:**
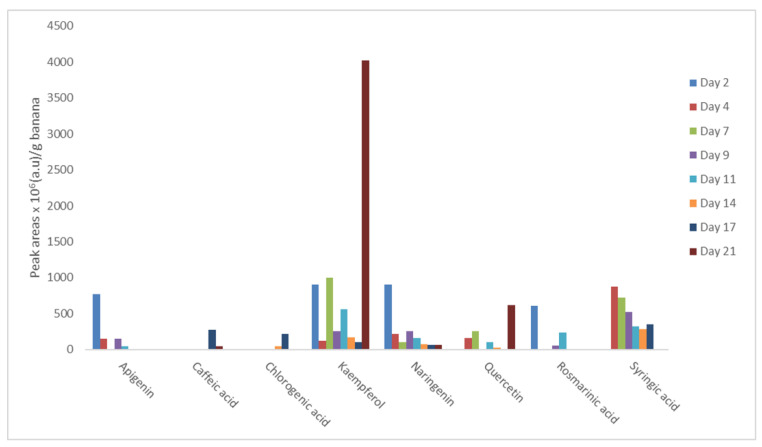
Variations in the content of phenolic compounds during ripening.

Similarly, rosmarinic acid, which was also determined from the start to the middle of ripening, was also elevated before ripening initiated. According to recent works [40], the detection of rosmarinic acid at the initial stages of ripening could be sustained, since this phenolic acid seems to delay the ethylene production and therefore the ripening process in climacteric fruits, such as tomatoes. Caffeic acid, a phenolic molecule which in other studies showed a very strong positive correlation with ripening [41] and was detected only in the ripe bananas (days 17 and 21), demonstrated a downward trend as the ripening progressed. On contrary, chlorogenic acid, which was also a possible index of banana ripening (days 14 and 17), since it was detected at the final stages of storage, showed an abrupt increase. According to Clifford et al. (2017) and Cai et al. (2019), chlorogenic acid increase, as a product of phenylalanine metabolism, can be recognized as an indicator for fruit ripening [16,42]. The same trend was also noticed in the case of quercetin. In relation to this observation, the increase of antiradical and antioxidant activities at day 11 until the end of the storage period could be correlated with quercetin detection at the same period. It is reported that quercetin, which is the soluble and bound flavonoid, acts as the free radicals’ scavenger and is associated with strong antioxidant activity [37,43]. Furthermore, it is reported that quercetin and kaempferol have a strong effect on cardiovascular protection [37]. Finally, syringic acid, which was not detected at the first (day 2) and at the last (day 21) day of storage, presented an increased content, which was progressively decreased in line with the observations of Shamla and Nisha (2017) [44]. Ultimately, the content of flavonoids was, in general, more elevated in banana extracts compared to phenolic acids. The variations in the concentration of all identified phenolics are illustrated in Figure 2.

In line with the results of other published studies [6,9,14], the presence of flavonoids and phenolic acids was enhanced when bananas were relatively unripe and then it was less prevalent as the fruit ripening advanced.

### 3.3. Computational Studies

**Figure 3:**
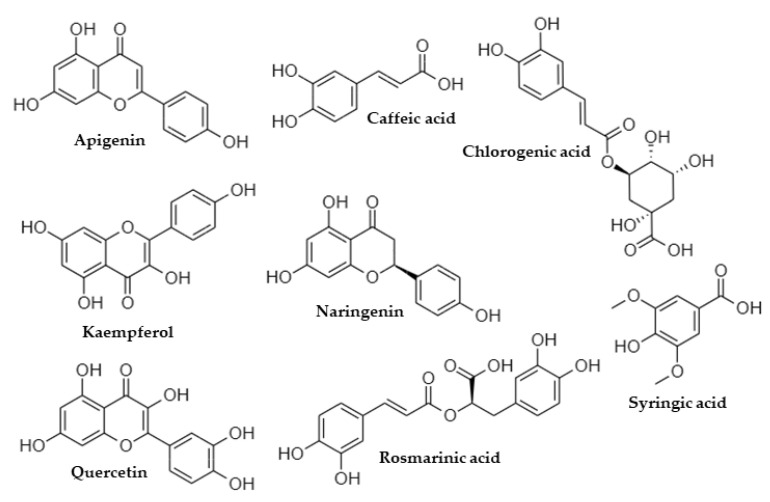
The chemical structures of the examined phytochemicals.

The phytochemicals that were identified, using LC-ESI(−)-MS/MS analysis, were generated (Figure 3) for further in silico studies, since besides their ripening-related properties, they are natural compounds with established beneficial health effects [45,46].

#### Target Prediction and Molecular Docking Studies

In an effort to correlate the chemical scaffolds of phenolics detected in banana samples with potential molecular targets and consequently with health benefits, the following open-web-based tools Random Forest QSAR (http://rfqsar.kaist.ac.kr/home.php) (accessed on 1 December 2022) and Swiss Target Prediction (http://www.swisstargetprediction.ch/) (accessed on 1 December 2022) [29] were used. The assessment of the results, based on probability values (Table S2, Supplementary Materials), pointed out human Carbonic Anhydrase II (hCA-II) and XII (hCA-XII) in consensus as the most promising molecular targets. Subsequently, the potential inhibitory activity of all investigated phytochemicals against the above-mentioned targets were examined through molecular docking studies. 

The suggested enzymes pertain to the Carbonic Anhydrase (CA) family which contains zinc-enzymes, that catalyze the reversible hydration of carbon dioxide (CO_2_) to bicarbonate ions (HCO_3_^−^) and protons [47]. This simple transformation is involved in a wide range of physiological processes (such as pH regulation, gas exchange, ion transport, bone resorption, fatty acid metabolism, etc.) [48]. In addition, CAs are associated with a plethora of pathological conditions, including glaucoma, edema, epilepsy, obesity, hypertension, cancer, neuropathic pain, and other neurological disorders [49,50].

Nowadays, the most common and well-established class of CA inhibitors (CAIs) are sulfonamides, such as acetazolamide, methazolamide, brinzolamide, etc., and their bioisosters (sulfamides and sulfamates). Although these compounds exhibit potent activity for CA enzymes, their lack of specificity may contribute to off-target CA inhibition and thus may lead to a variety of side effects, from nausea and fatigue to depression. Therefore, the identification of novel scaffolds which overcome these drawbacks is at the forefront of medicinal chemistry [51,52]. Thereby, in the last decades, phenolic compounds have gained a prominent position as an alternative for the discovery of CAs inhibitors [53,54,55].

Generally, molecular docking results analysis at the binding site of hCA-II (PDB ID: 5AML) and hCA-XII (PDB ID: 5MSA) indicated that the examined phytochemicals possessed similar or greater binding energy values compared to the co-crystallized sulfonamides (Table 5), respectively. Moreover, the docking poses assessment was based not only on docking scores but also on the presence of interactions with the crucial amino acids determined by the co-crystallized ligands of the examined enzymes. It is critical to highlight that the visual inspection of the derived poses revealed that all tested compounds were stabilized into the binding pocket of hCA-II and hCA-XII enzymes forming metal-coordination with the zinc atom. The interaction pattern of the sulfonamides and examined phenolic compounds for each enzyme target is illustrated in Table 6. 

Regarding the hCA-II enzyme, the docking scores of the phenolic compounds display higher values in almost all cases compared to the co-crystallized sulphonamide. Furthermore, a hydrogen bond with the crucial amino acid Thr199 and a metal coordination were also observed, highlighting their potential to inhibit the enzyme. Among the tested compounds, chlorogenic acid and rosmarinic acid present a common interaction motif, forming hydrogen bonds not only with the crucial amino acids (Gln92 and Thr199), but also with Trp5, Asn67, and Pro201, providing a strong indication for their binding into hCA-II enzyme. Likewise, the docked pose of naringenin, a phenolic compound that was presented during all stages of banana ripening, indicated interesting results. Apart from the metal-coordination with the zinc atom, especially, it presents a variety of interactions, consisting of hydrogen bonds and pi–pi interactions, reinforcing this way its potential binding affinity. 

The docked poses of the phenolic compounds at the binding site of hCA-XII showed a significantly higher binding affinity for all compounds compared to the co-crystallized ligand. In addition, all compounds create metal-coordination with the zinc atom and/or also develop a hydrogen bond with the crucial amino acid Thr198. These facts create a strong indication that all examined phytochemicals could potentially inhibit hCA-XII enzyme. Among tested compounds, rosmarinic acid may exhibit a strong binding capacity because its binding is enhanced through the formation of hydrogen bonds with Trp4, Gln89, and Pro200 amino acids. 

**Figure 4:**
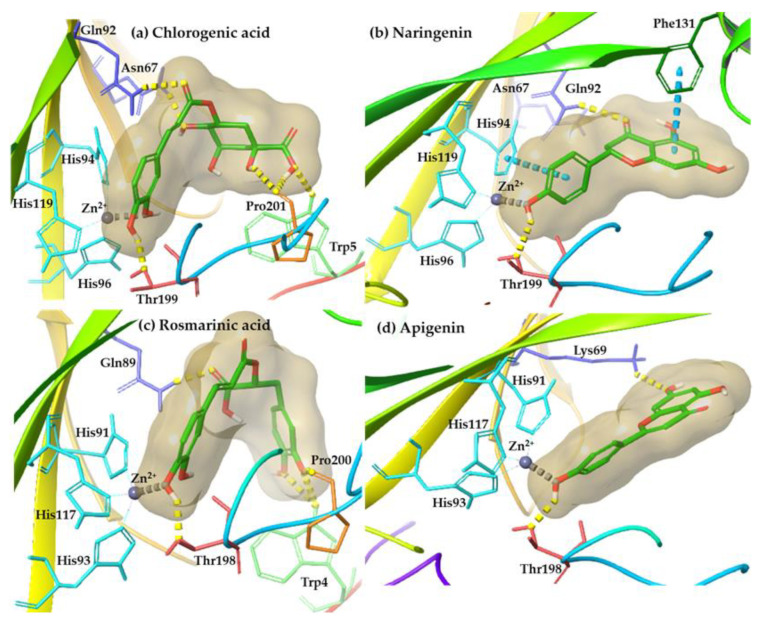
Representative binding poses of (**a**) chlorogenic acid and (**b**) naringenin at hCA-II enzyme and (**c**) rosmarinic acid and (**d**) apigenin at hCA-XII, derived from molecular docking studies. Metal coordination, hydrogen bonds, and pi–pi interaction are depicted with grey, yellow, and blue dashed lines, respectively.

To conclude, the molecular docking results of phenolic compounds into the binding site of hCA-II and hCA-XII enzymes revealed that all compounds form favorable interactions and may contribute to the discovery of novel CA inhibitors. Representative binding poses of phenolic compounds at the binding site of hCA-II and hCA-XII are presented in Figure 4.

## 4. Conclusions

In this context, the objective of the current study was to evaluate the bioactive content of bananas in terms of its effect on banana ripening and its potential against various pathologies. As indicated by the results, the TPC of banana pulp was decreased during fruit maturation and then exhibited an exponential increase at the last stage (days 17 and 21) of ripening, which could be assigned to the release of the conjugated phenolic molecules or to their production via certain metabolic pathways (i.e. phenylalaline metabolism). A similar but smoother trend (decreased and then increased till stabilization values) was also recorded in the antioxidant and antiradical activities of bananas extracts. The phenolic fingerprint, provided by LC-MS/MS analysis, confirmed the presence of various phenolic acids and flavonoids. Among them, kaempferol and naringenin was detected in all stages of ripening, but in different contents according to the day of storage. For instance, naringenin was decreased during ripening, while kaempferol showed an immense increase at the last day of storage. Apigenin and rosmarinic acid could be considered as representative phenolics of fresh bananas, while quercetin, caffeic, and chlorogenic acid could be acknowledged as indicators of bananas ripening.

The in silico studies, which were performed by applying molecular target prediction tools, highlighted human carbonic anhydrase II (hCA-II) and XII (hCA-XII) enzymes, related to several pathologies (hypertension, metabolic syndromes, edema, etc.) as potential targets of the identified phytochemicals.

Therefore, the assessment of phenolic content of banana or other fruits could provide further information regarding the ripening of the fruit under study and could pave the way for proposing new candidates which may serve as suitable templates with a putative inhibitory activity against various enzymes. 

## Figures and Tables

**Table 1:** Gradient elution program for the separation of phenolic compounds.

Steps of Elution (min)	Water-0.2% *v*/*v* Formic Acid	Acetonitrile-0.1% *v*/*v* Formic Acid	Flow Rate (μL/min)
0.0	90	10	300
0.5	80	20	300
4.0	70	30	300
4.1	50	50	350
4.5	50	50	350
5.1	35	65	350
7.0	0	100	350
8.0	0	100	350
9.0	0	100	300
9.1	90	10	300
15	90	10	300

**Table 2:** Pairwise correlation matrix among spectrophometric assays of banana.

Correlation Coefficients	TPC	ABTS^●+^	FRAP
TPC	1	0.53	0.52
ABTS^●+^	0.53	1	0.95
FRAP	0.52	0.95	1

^●+^ the symbol of the positevely charged stable radical of ABTS.

**Table 3:** Chromatographic and mass spectral information of the phenolics standards.

Phenolic Compound	Retention Time (min)	Parent Ion [M-H]^−^	Product Ions (MS/MS)
Apigenin	6.60	269.1	151.4, 149.3, **117.2** *
Caffeic acid	2.75	179.1	**135.4** *, 107.3
Chlorogenic acid	1.72	353.2	**191.5** *, 179.6, 161.5
Kaempferol	6.66	285.3	**257.6** *, 229.6, 187.5
Naringenin	6.64	271.1	177.4, 151.4, **119.3** *, 107.3, 93.2
Quercetin	6.20	301.1	273.5, 179.3, **151.4** *
Rosmarinic acid	4.95	359.3	197.5, 179.5, **161.5** *, 135.4
Syringic acid	2.62	197.0	**182.5** *, 167.5, 123.2, 95.2

* The fragments in bold are the most intense MS/MS fragments and were used for the peak area quantification of each phenolic compound.

**Table 4:** Phenolic compounds identified at different days of ripening process.

Storage Period (Days)	Apigenin 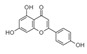	Caffeic Acid 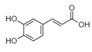	Chlorogenic Acid 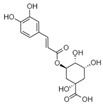	Kaempferol 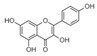	Naringenin 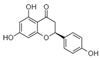	Quercetin 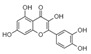	Rosmarinic Acid 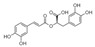	Syringic Acid
2	√			√	√		√	
4	√			√	√			√
7				√	√			√
9	√			√	√		√	√
11	√			√	√	√	√	√
14			√	√	√	√		√
17		√	√	√	√			√
21		√		√	√	√		

**Table 5:** Docking scores and distance from the zinc atom of the examined compounds and the co-crystallized inhibitors at the binding site of the hCA-II and hCA-XII enzymes.

Phenolic Compounds	hCA-II		hCA-XII	
	Docking Score (kcal · mol^−1^)	Distance Zn^2+^ (Å)	Docking Score (kcal · mol^−1^)	Distance Zn^2+^ (Å)
2-(But-2-yn-1-ylsulfamoyl)-4-sulfamoylbenzoic acid	−4.86	1.76	^1^ NT	NT
2,3,5,6-Tetrafluoro-4-(propylthio)benzenesulfonamide	NT	NT	−3.67	1.76
Apigenin	−5.70	2.23	−5.64	2.01
Caffeic acid	−4.46	2.05	−5.26	2.12
Chlorogenic acid	−5.73	2.06	−5.95	2.17
Kaempferol	−5.31	2.06	−4.90	2.04
Naringenin	−5.32	2.05	−5.02	2.00
Quercetin	−5.13	2.23	−5.29	2.17
Rosmarinic acid	−4.72	2.05	−5.73	2.12
Syringic acid	−5.48	2.05	−4.89	2.15

^1^ NT: Not Tested.

**Table 6:** The interaction patterns of co-crystallized ligands and examined phenolic compounds at the binding sites of hCA-II and hCA-XII enzymes. The common interactions among the tested compounds and co-crystallized ligands are marked in bold font.

Phenolic Compounds	hCA-II (PDB ID: 5AML)	hCA-XII (PDB ID: 5MSA)
	Interactions	
2-(But-2-yn-1-ylsulfamoyl)-4-sulfamoylbenzoic acid	**^1^** HB: **Gln92, Thr199** & ^2^ **mc**	
2,3,5,6-Tetrafluoro-4-(propylthio)benzenesulfonamide		HB: **Thr198** & **mc**
Apigenin	HB: **Thr199** & **mc**	HB: Lys69, **Thr198** & **mc**
Caffeic acid	HB: **Thr199,** Asn67 **& mc**	HB: **Thr198** & **mc**
Chlorogenic acid	HB: Trp5, Asn67, **Gln92**, **Thr199**, Pro201 & **mc**	HB: Thr88, Gln89 & **mc**
Kaempferol	HB: **Thr199** & **mc**	**mc**
Naringenin	HB: **Gln92**, His64, **Thr199** & pi-pi Phe131 & **mc**	HB: **Thr198** & **mc**
Quercetin	pi-pi: His94, Phe131 & **mc**	HB: **Thr198**, Thr199 & **mc**
Rosmarinic acid	HB: Trp5, Asn67, **Gln92**, **Thr199**, Pro201 & **mc**	HB: Trp4, Gln89, **Thr198**, Pro200 & **mc**
Syringic acid	HB: **Thr199**, Thr200 & pi-pi His94 & **mc**	HB: Gln89, **Thr198** & **mc**

^1^ HB: Hydrogen Bond; ^2^ mc: metal-coordination.

## Data Availability

Not applicable.

## Supplementary Materials

The following are available online at https://www.mdpi.com/article/10.3390/life13020332/s1, Table S1: Parameters of EMS and IDA experiments Table S2: Molecular target prediction probability values of examined phenolic compounds, derived from the application of open-web-based tools Random Forest QSAR (http://rfqsar.kaist.ac.kr/home.php) (accessed on 1 December 2022) and Swiss Target Prediction (http://www.swisstargetprediction.ch/) (accessed on 1 December 2022); Table S3. Results of total phenolic content (TPC), antiradical (ABTS), and antioxidant activity (FRAP) of banana flesh samples during storage at 18.0 ± 0.5 °C; Figure S1: Representative chromatographs and EMS mass spectra of indicative elucidated compounds. (a) rosmarinic acid; (b) kaempferol; (c) quercetin.
