# Supplementary material for: Potential Health Benefits of Banana Phenolic Content during Ripening by Implementing Analytical and In Silico Techniques
Source: Life (Basel). 2023 Jan 25;13(2):332. doi: 10.3390/life13020332 (PMC9962436; doi:10.3390/life13020332)
Supplement: Supplementary file 1 [file life-13-00332-s001.zip › life-2141422-supplementary.pdf]

Optimization of MS parameters

Flow injection analysis (FIA) of 10 µg/mL from each compound was conducted for source optimization. The parameters for EMS and IDA experiments are mentioned above.

Table S1. Parameters of EMS and IDA experiments.

Parameters	EMS	IDA-EPI
Mass range (m/z)	100-700	50-700
Scan period (s)	0.1513	
Number of scans	3	1
Scan rate (amu/s)	4000	1000
Curtain gas	30	30
CAD gas	Medium	High
Temperature (°C)	550	550
Gas 1	45	45
Gas 2	45	45
Ion spray	-4500	-4500
Declustering potential	-30	-30
Entrance potential	-7.5	-7.5
Collision energy	-10	-30
C2B	-300	-450
IPA threshold for EPI experiments		10000 counts
Peak selected for the EPI scan		Two most intense peaks
Q1 resolution		Low
Collision energy spray		10

Table S2. Molecular target prediction probability values of examined phenolic compounds, derived from the application of open web-based tools Random Forest QSAR (http://rfqsar.kaist.ac.kr/home.php) (accessed on 1 December 2022) and Swiss Target Prediction (http://www.swisstargetprediction.ch/) (accessed on 1 December 2022).

Compounds	Probability Value Random Forest QSAR		Probability Value Swiss Target Prediction	
	hCA-II	hCA-XII	hCA-II	hCA-XII
Apigenin	0.74	0.75	0.56	0.56
Caffeic acid	0.80	0.82	0.74	0.74
Chlorogenic acid	0.05	-	0.10	0.10
Kaempferol	0.83	0.83	1.00	1.00
Naringenin	-	0.80	0.10	0.91
Quercetin	0.83	0.84	1.00	1.00
Rosmarinic acid	0.65	0.69	0.17	0.12
Syringic acid	0.80	0.82	1.00	1.00

Table S3. Results of total phenolic content (TPC), antiradical (ABTS) and antioxidant activity (FRAP) of banana flesh samples during storage at 18.0±0.5 °C.

Days	Total Phenolic Content (TPC) (mg GAE / g of banana flesh)	Antiradical activity (ABTS) (mg TE / g of banana flesh)	Antioxidant activity (FRAP) (mg Fe(II) / g of banana flesh)
2	0.309±0.063a	0.971±0.080a	2.846±0.275a
4	0.157±0.053bc	0.405±0.070b	1.507±0.273be
7	0.118±0.020b	0.385±0.036b	1.195±0.105c
9	0.122±0.027bc	0.407±0.036b	1.324±0.121cb
11	0.152±0.020bc	0.408±0.047b	1.416±0.147cbd
14	0.180±0.026c	0.444±0.024bc	1.620±0.127de
17	0.384±0.037d	0.500±0.041c	1.649±0.166de
21	0.467±0.043e	0.578±0.061d	1.762±0.196e

a-e: Different letters in the results of each analysis indicate that the values of the samples differ significantly.

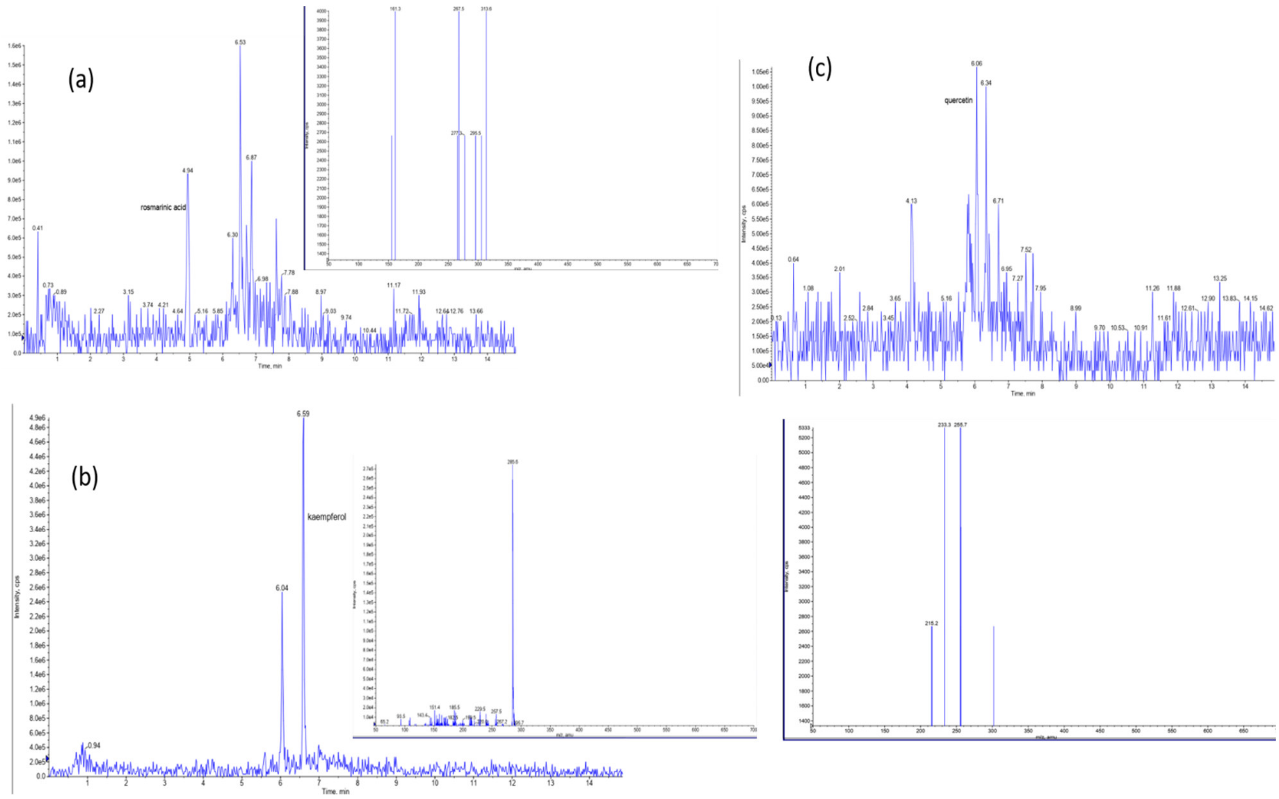

Figure S1. Representative chromatographs and EMS mass spectra of indicative elucidated compounds. (a) rosmarinic acid; (b) kaempferol; (c) quercetin.
